# Effects of agronomical practices on potato growth, nutritional profile, and suitability for frying

**DOI:** 10.1002/jsfa.14147

**Published:** 2025-01-30

**Authors:** Francesca Bruno, Ingo Hein, M Ehsan Jorat, Moira Ledbetter, Brian Harrower, Ben Davies, Keith Sturrock, Gary Montague, Malcolm Knott, Ged McNamara, Alberto Fiore

**Affiliations:** ^1^ Department of Built Environment and Life Sciences, Faculty of Social and Applied Sciences University of Abertay Dundee UK; ^2^ The James Hutton Institute Dundee UK; ^3^ KP Snacks Billingham UK; ^4^ Division of Psychology and Forensic Science School of Applied Sciences, University of Abertay Dundee UK; ^5^ School of Science, Engineering and Design, Teesside University Middlesborough UK; ^6^ Industrial Technology Systems, ITS House Middlesbrough UK

**Keywords:** acrylamide, potato crisps, sulfur fertilization, basalt rock aggregate, speed breeding, LED lights

## Abstract

**BACKGROUND:**

This study investigated the effect of sulfur nutrition, basalt rock aggregate (BA) application, with a carbon capture function, and speed breeding under light‐emitting diode (LED) light, on the nutritional profile of potatoes and acrylamide formation in crisp production.

**RESULTS:**

Taurus potatoes grown with sulfur showed reduced glucose, sucrose, and total amino acids, and increased asparagine. No difference in acrylamide content was observed in crisps from Taurus and Lady Claire cultivars, with either sulfur or BA application. Speed breeding under LED light reduced plant height in all cultivars (50–60 cm) in comparison with controls (80–90 cm). Tubers grown under LED light exhibited higher levels of glucose and fructose, and increased formation of acrylamide in crisps (78.90% for Lady Claire, 592.58% for Taurus, and 70.25% for Desiree).

**CONCLUSIONS:**

Sulfur fertilization could benefit certain potato cultivars by lowering sugar levels in the tubers. Basal rock aggregate can be applied safely during the growth of potatoes as an innovative tool for sequestering carbon dioxide (CO_2_) from the atmosphere, with no negative effect on tubers’ nutritional profile and no influence on acrylamide formation in crisps. The LED light conditions proved to be unsuitable for potato growth, especially if the potatoes were destined for the frying industry, increasing both sugars and acrylamide content. © 2025 The Author(s). *Journal of the Science of Food and Agriculture* published by John Wiley & Sons Ltd on behalf of Society of Chemical Industry.

## INTRODUCTION

Potato (*Solanum tuberosum* L.) is the fourth most important food crop after wheat, rice, and maize, with a total world production of 375 million t in 2022.^1^ It is a very diverse and adaptable crop with strong yield and high nutritive value.^2^ Potato products are very susceptible to the formation of acrylamide – a processing contaminant classified as probable carcinogen.^3^, ^4^ Acrylamide is mainly formed during the Maillard reaction in foods containing reducing sugars and amino acids when they are cooked at temperatures over 120 °C, typically during baking, frying, or roasting.^5^, ^6^, ^7^, ^8^, ^9^, ^10^ Potato tubers contain high amounts of acrylamide precursors, in particular asparagine, glucose, and fructose; potato products such as fried potato crisps and chips are therefore among the main sources of acrylamide in the Western diet.^11^, ^12^, ^13^, ^14^, ^15^ Much research has been conducted to investigate possible ways to reduce acrylamide formation, with potato products being one of the most commonly studied food matrices.^5^, ^6^, ^16^ However, most mitigation strategies available to reduce the contaminant levels are difficult to apply in large‐scale manufacturing due to associated costs, because they are time consuming, or because they are not as effective as on a laboratory scale.^17^ The aim of this study was to explore selected agronomic practices as possible cost‐effective approaches, by investigating their effect on potato growth and acrylamide‐forming potential.

### Sulfur nutrition

Nitrogen and sulfur are the most common minerals applied to potatoes as soil fertilizers. With regard to the formation of acrylamide, several studies have been conducted to investigate how the levels of soil nutrients can influence crop composition and consequently food quality and safety.^7^, ^18^, ^19^, ^20^, ^21^, ^22^, ^23^ Nitrogen supply seems to decrease sugar levels in potato tubers; however, the effect is variety dependent and sometimes this change is not observed.^21^, ^23^, ^24^ On the other hand, nitrogen nutrition tends to increase the levels of asparagine, which has a higher N:C ratio.^22^, ^24^ Increased asparagine biosynthesis could explain the effect on lowering sugar levels.^22^, ^24^ The influence of nitrogen fertilization on acrylamide forming potential was also found to be cultivar dependent, with either an increase in contaminant formation or no significant effect on it.^7^, ^21^, ^23^

Sulfur deprivation was found to increase sugars levels in all varieties tested, in a glass‐pot trial conducted by Elmore *et al*.^19^, ^20^ Total free amino acids were also higher in tubers from sulfur‐deprived plants, but the main amino acids accumulated changed within different varieties. However, acrylamide formation was found to be always lower in potatoes grown with sulfur deprivation, possibly because sugars could react with any of the other amino acids in the tubers, reducing the levels of sugars available to interact with asparagine and form acrylamide.^19^, ^20^ In a subsequent field study conducted by Muttucumaru *et al*.,^21^ sulfur nutrition was found to have no effect on free asparagine concentration and to decrease glucose levels in the tubers. Sulfur fertilization also seemed to mitigate the effect of nitrogen application in increasing the acrylamide‐forming potential of French fries and was able to mitigate acrylamide formation by 27%.^21^

Considering results from the studies mentioned above, a field trial was designed in the current study to investigate the effects of sulfur fertilization on the nutritional profile of potatoes, including acrylamide precursors, and on the acrylamide formation in potato crisps. For this purpose, two potato cultivars, Lady Claire and Taurus, commonly used to produce crisps, were selected.

### Basalt rock aggregate application

Research has shown that soils containing volcanic basic silicate rocks, such as dolerites or basalt, can remove substantial amounts of CO_2_ inorganically from the atmosphere in a permanent and stable form, through the formation of soil carbonates in soil.^25^, ^26^, ^27^ This is an innovative technique for sequestering CO_2_, which considers both climate change and plant health. Silicate minerals, in particular calcium silicates, which occur commonly in basic igneous rocks such as basalt and dolerites, react with dissolved CO_2_ in soil derived from photosynthesis to form carbonates.^28^ The strong bonds between the chemical elements that compose silicate rocks result in a gradual release of the elements, which can span geological time scales. Plants and microorganisms are capable of influencing release of the elements through their action on mineral surfaces, which are controlled by their living requirements.^27^, ^29^ The natural chemical weathering of volcanic silicate rocks, which occurs on geologic timescales, is a process that regulates atmospheric CO_2_. This can be accelerated by applying silicate rocks to the land surface in what is called ‘enhanced weathering’.^30^, ^31^, ^32^ Basic silicate rocks are abundant in the UK with a high concentration in Scotland. Silicate rock quarry is a by‐product of standard quarry crushing and milling processes and represents almost the totality of waste generated by the quarrying industry.^33^ Currently the aggregates below 4 mm in diameter remined from quarry crushing and milling processes are referred to as a by‐product as they have minimal use in construction industry which is the main destination for basic silicate rock aggregates. However, this aggregate size is an ideal size for use in agricultural lands.^34^, ^35^, ^36^ Growing edible plants using basic silicate rock, which provides an additional CO_2_ capture function, is becoming acceptable and examples can be seen in small to large‐scale applications.^27^, ^37^, ^38^ Basalt rock aggregates (BA) are currently available commercially as soil and compost remineralizer for food‐growing purposes, which can help boost fertility and yield.

X‐ray fluorescence (XRF) testing of three dolerite samples showed large amounts of elements such as silicon oxide, which supports plant tissue development, iron and manganese oxides (which are plant micronutrients), and calcium, magnesium and potassium oxides (which are plant macronutrients).^34^ Although a strong body of research exists on basic silicate rock weathering in soil and associated impacts, particularly in urban soil, its impact on plants and products when the basalt or dolerite are added to agricultural fields are unknown.

Two pot trials were designed to understand the influence of BA application on the growth of potato plants and to examine the impact on the nutritional quality and safety of tubers. The study also focused on eventual changes in acrylamide precursor levels in the tubers and acrylamide formation in generated potato crisps to ensure that BA application is suitable for potatoes destined to the processing market and for the production of fried potato products.

### Growth in LED light glasshouse

Traditional soil‐based agriculture is currently facing several challenges. Global climate change, urbanization, and the use of chemicals and pesticides have deeply and negatively affected the fertility and productivity of the soil.^39^, ^40^, ^41^, ^42^ The reduction in crop productivity and the decline in cultivable land, together with environmental factors such as increasing temperatures, uncertain meteorological conditions, dry spells, and limited availability of water for irrigation, are all a threat to food production and food security.^42^, ^43^, ^44^ Indoor cultivation systems, such as vertical farming systems, represent a promising alternative to soil‐based farming, which is becoming increasingly popular due to its many advantages. As closed systems, they allow plants to be grown without the use of pesticides; all climate factors are controlled, and the ideal conditions of lighting, humidity, and temperature can be applied, which results in increased yields and productivity.^40^, ^43^, ^44^, ^45^ Light‐emitting diode (LED) lights are being applied increasingly as light sources for indoor cultivation systems where artificial light is employed, due to their advantages over other commonly used light sources. Light‐emitting diodes have high energy conversion efficiency with low power consumption, a long lifetime, and low thermal output; they offer a high level of control over light spectrum and intensity, with the possibility of selecting specific wavelengths for targeted responses. They are also very compact and portable, and controls can be integrated easily into electronic systems.^46^, ^47^, ^48^, ^49^ Wavelength, intensity and duration of light are all key factors that influence plant growth and development, as well as crop production. Light drives photosynthesis and so regulates all main aspects from seed propagation to leaf structure and number, plant height, root length, flowering, fruiting, synthesis, and the accumulation of metabolites and phytochemicals.^47^, ^48^

The aim of this work was to investigate the effect of irradiation with LED lights during plant growth on the levels of acrylamide precursors, reducing sugars and asparagine, and more generally amino acidic and sugar profile in potato tubers; as well as the resulting influence on acrylamide formation during the frying of potatoes to produce crisps.

## MATERIALS AND METHODS

### Chemicals and reagents

Methanol (LC–MS grade), water (LC–MS grade), acetonitrile (HPLC grade), hexane (HPLC grade), sodium chloride (NaCl, 99.5%) and pyridine anhydrous (99.5%) were purchased from Fisher Scientific (Loughborough, UK). Magnesium sulfate (MgSO_4_, 97%) was purchased from Acros Organics (Geel, Belgium). Primary secondary amine sorbent (PSA) was purchased from Agilent Technologies (Santa Clara, CA, USA). Acrylamide (98%) was purchased from Fluka (Buchs, Switzerland). [2,3,3‐d^3^]‐acrylamide (98%), formic acid (liquid chromatography–mass spectrometry (LC–MS) grade), and cycloleucine (97%) were purchased from Sigma‐Aldrich (Gillingham, UK). *N*‐Methyl‐*N*‐(trimethylsilyl) trifluoroacetamide (MSTFA) (100%) was purchased from Fluorochem (Hadfield, UK).

### Food material

Lady Claire, Taurus, and Desiree variety potatoes were grown at James Hutton Institute (JHI) in Dundee, UK. Palm oil (RSPO Palm RD Oil) was purchased from Kerfoot Oil Specialists (Northallerton, UK). Remin Basalt Rock Aggregate was purchased from Agralan Ltd (Swindon UK).

### Growth conditions

#### Sulfur nutrition

A field trial was set up at the JHI where potato plants were grown with and without the addition of sulfur to the soil (25 kg of SO_3_ per ha). Two potato cultivars were trialled – Lady Claire and Taurus. The design (Supporting Information, Fig. S1) consisted of a total of 24 plots, six replicates plots for each of the four conditions (Lady Claire with sulfur, Lady Claire control, Taurus with sulfur, and Taurus control); the treatment of the plots was fully randomized. Each plot consisted of four drills with 16 plants per drill – a total of 64 plants per plot. The two exterior drills plus four first rows and two last rows were used as buffer plants surrounding the experimental material, with only 20 plants in total per plot harvested to be assessed (Supporting Information, Fig. S1). Plants were planted on the 1 May and harvested after 16 weeks at the end of August. Light and temperature conditions varied during the growing season according to weather variations.

#### Basalt rock aggregate

Two trials were set up at JHI where plants were grown in pots in an unheated glasshouse, with and without addition of BA. Two potato cultivars were trialled, Lady Claire and Taurus. Light and temperature conditions varied according to growing season and weather variations. In the first trial a total of 100 plants (50 of Lady Claire variety and 50 of Taurus variety) were planted on 10 August 2020; 50 g per pot of BA was added to half of the plants (25 from Lady Claire and 25 from Taurus) on three occasions by mixing it with the soil, with the remaining plants used as controls. Potatoes were harvested after 12 weeks, almost 1 month earlier than usual, on 27 October 2020 due to the low temperatures starting to affect the plants. As with the first trial, for the second trial 100 plants were planted on the 1 April 2021, 50 plants for each variety; 50 g per pot of BA was added to half of the plants on three occasions by mixing it with the soil. Potatoes were harvested after 16 weeks on 22 July 2021.

#### Speed breeding under LED lights

A speed‐breeding trial was set up at the JHI, in which plants were grown under high‐intensity light conditions for 22 h per day. This has been shown to accelerate plants’ life cycles, leading to faster flowering and tuberization.^44^, ^46^, ^50^ Three potato cultivars were trialed: Lady Claire, Taurus, and Desiree. Twelve tubers for each variety were planted on 1 April 2021 in a heated glasshouse and grown under LED lighting (Elixia LED lamps, Heliospectra, Gothenburg, Sweden) according to the Nature protocol from Ghosh *et al*.^50^, ^51^ The plants were shielded from natural sunlight by covering the glass with reflective insulating material. Light and temperature conditions were as follows: 22 h of light from 01:00 a.m. to 11:00 p.m. at 18 °C; 2 h of darkness from 11:00 a.m. to 01:00 a.m. at 18 °C. Spectrum readings with specifications on LED lighting parameters are included in the supporting information (Supporting Information, Figs [Link], [Link]). Similarly, on the same date, 12 control tubers for each variety were planted in a heated glasshouse and grown under natural sunlight supplemented by sodium glasshouse lighting. Key indicators of plant growth and lifecycle were measured throughout the experiment. Potatoes from control plants were harvested after 20 weeks from planting; potatoes grown under LED lights were harvested after 14 weeks from planting for the Lady Claire and Desiree cultivars, and after 16 weeks from planting for the Taurus cultivar.

### Sampling and crisp production

For all the trials (sulfur trial, first and second BA trials, LED glasshouse trial) potato tubers were processed within a day after harvest. Harvesting and sampling was as follows:

- Sulfur trial: potatoes were collected in six bags per condition, each bag corresponding to a plot (64 bags in total) and containing tubers from 20 plants. From four of the six bags per condition, six tubers per bag were randomly selected to give a total of *n* = 24 potato samples per condition that were processed and fried to generate potato crisps.
- First BA trial: potatoes were collected in four different bags per condition, giving a total of 16 bags, each bag containing potatoes from more than one plant. Six tubers from each bag were selected randomly to give a total of *n* = 24 potato samples per condition, which were processed and fried to generate potato crisps.
- Second BA trial: as above, potatoes were collected in four bags per condition giving a total of 16 bags, each bag containing potatoes from more than one plant. From two of the four bags, 6 tubers were randomly selected from each bag to give a total of *n* = 12 potato samples per condition, which were processed and fried to generate potato crisps.
- LED light glasshouse trial: potatoes were collected in 1 bag per condition, giving a total of 6 bags (Lady Claire controls, Lady Claire LED lights, Taurus controls, Taurus LED lights, Desiree controls, Desiree LED lights), each bag containing potatoes from more than one plant. From each bag *n* = 12 tubers were randomly selected, processed, and fried.

When frying could not be completed within the same day, due to sample size, potatoes were stored in a cold room with controlled humidity and temperature at 8–10 °C while awaiting processing to avoid cold sweetening.

Potatoes were washed and manually sliced using Food and Meat (FAM) Urschel slicer blades with a 0.212 V‐shaped cut and a 0.80 mm shim (Urschel Laboratories, Leicester, UK). A 30 mm disc was taken from the slices. Potato slices (20 g) were soaked in 2 L of distilled water for 2 min at room temperature, then immersed in 2 L distilled water at 78 °C for 3 min. Samples were then withdrawn and excess water was drained.

The samples were fried in palm oil at 173 ± 2 °C in a 3 L Selection Magimix professional deep fat fryer (Godalming, UK). Commercial processing conditions were adopted from Bartlett *et al*.,^52^ with some modification, frying time was 4.5 min. The oil temperature was monitored by an external probe (Electronic Temperature Instruments LTD, Worthing, UK). Potato crisps were removed from the fryer, drained to remove the excess oil, and cooled, then pulverized and stored at −18 °C until analysis.

### Acrylamide quantification

Acrylamide content in the generated potato crisps was quantified by liquid chromatography tandem‐mass spectrometry (LC‐MS/MS) using a three‐phase extraction method as described by Bruno *et al*.^53^

Acrylamide quantification was performed on Thermos Fisher Scientific LC‐MS/MS equipment (San Jose, CA, USA) consisting of a degasser, a quaternary pump, a thermostatic autosampler, a column oven, and a TSQ mass spectrometer equipped with an electrospray ionization (ESI) source.^53^

### Metabolomic analysis of raw material

Samples of raw potatoes collected prior to washing, before frying, were analyzed using (GC–MS) to determine the metabolomic profile of the tubers, as described by Bruno *et al*.^53^ Acrylamide precursors (glucose, fructose, asparagine), as well as sucrose and other amino acids, were quantified.

### Statistical analysis

Statistical analysis was using Jamovi computer software, version 2.5. The Shapiro–Wilk test was used to check normality of the data with an *α* value of 0.05 taken as significant. A one‐way analysis of variance (ANOVA) was performed to show significant differences between samples at the *P* < 0.05 confidence level. A Tukey test was performed as a post hoc test with a one‐way ANOVA to identify differences between groups.

## RESULTS AND DISCUSSION

### Sulfur nutrition

Sulfur fertilization of the soil resulted in potatoes with lower average weights for the cultivar Lady Claire (179.6 ± 29.3 g compared to 201.5 ± 30.3 g in controls), and higher average weights for the cultivar Taurus (183.4 ± 18.4 g compared to 171.6 ± 18.9 g in controls) (Supporting Information, Table S5).

Table 1 reports the average sugar and amino acid content of potatoes grown with and without a sulfur supply.

**Table 1:** Sugar and amino acidic profile of raw potato tubers grown with and without sulfur nutrition^a^

Variety	Lady Claire		Taurus	
Treatment^b^	Control	Sulfur	Control	Sulfur
Glucose	1.33 ± 0.33 a	1.56 ± 0.61 a	6.22 ± 3.36 a	3.18 ± 1.56 b
Fructose	0.52 ± 0.24 a	0.58 ± 0.30 a	1.85 ± 0.81 a	1.80 ± 0.80 a
**Total reducing sugars**	**1.92 ± 0.69 a**	**2.21 ± 0.93 a**	**7.34 ± 3.57 a**	**5.42 ± 2.74 a**
Sucrose	8.63 ± 3.76 a	9.68 ± 4.84 a	14.75 ± 8.47 a	7.98 ± 2.60 b
Asparagine	1.88 ± 1.04 a	1.88 ± 1.52 a	0.90 ± 0.45 a	2.95 ± 1.90 b
Alanine	1.28 ± 0.37 a	1.25 ± 0.57 a	0.90 ± 0.30 a	0.57 ± 0.26 b
Valine	3.39 ± 0.73 a	3.40 ± 1.27 a	4.45 ± 1.48 a	2.31 ± 0.59 b
Isoleucine	1.02 ± 0.25 a	1.58 ± 0.77 b	2.63 ± 1.11 a	1.27 ± 0.32 b
Glycine	0.99 ± 0.08 a	0.96 ± 0.25 a	1.44 ± 0.96 a	0.71 ± 0.20 a
Serine	0.88 ± 0.11 a	1.23 ± 0.70 a	1.69 ± 0.66 a	0.68 ± 0.14 b
Threonine	1.54 ± 0.24 a	1.67 ± 0.59 a	2.29 ± 0.71 a	1.49 ± 0.52 b
Lysine	0.93 ± 0.34 a	1.03 ± 0.48 a	1.12 ± 0.44 a	0.84 ± 0.23 b
Tyrosine	1.40 ± 0.65 a	1.33 ± 0.60 a	1.24 ± 0.51 a	1.09 ± 0.56 a
Tryptophan	0.84 ± 0.46 a	0.90 ± 0.44 a	0.81 ± 0.29 a	0.98 ± 0.52 a
**Total amino acids**	**13.16 ± 3.04 a**	**14.45 ± 4.26 a**	**15.04 ± 5.20 a**	**12.60 ± 4.08 a**

^a^ Concentrations are expressed in μgmg^−1^ cycloleucine equivalent. Results are expressed as means ± SDs, *n* = 24.

^b^ Different letters in the same row indicate significant differences (*P* < 0.05) between treated samples and controls within the same cultivar.

For tubers of the Lady Claire cultivar, compared with controls, no significant difference in sugars or amino acid content was observed due to sulfur addition to the soil, except for a higher isoleucine content. Low levels of both reducing sugars were measured overall. The average glucose content was 1.56 ± 0.61 μg mg^−1^ and 1.33 ± 0.33 μg mg^−1^ in potatoes grown with sulfur and controls respectively; the average fructose content was 0.58 ± 0.30 μg mg^−1^ and 0.52 ± 0.24 μg mg^−1^. For the Taurus cultivar, sulfur nutrition led to potatoes with significantly lower glucose (3.18 ± 1.56 μg mg^−1^) and sucrose (7.98 ± 2.60 μg mg^−1^) than controls (6.22 ± 3.36 μg mg^−1^ glucose and 14.75 ± 8.47 μg mg^−1^ sucrose). No difference was observed in fructose levels (1.80 ± 0.80 μg mg^−1^ with sulfur and 1.85 ± 0.81 μg mg^−1^ in controls). Lower alanine, valine, isoleucine, serine, threonine, lysine, but higher asparagine, were measured in Taurus raw potatoes from plants grown with sulfur added to the soil.

**Figure 1:**
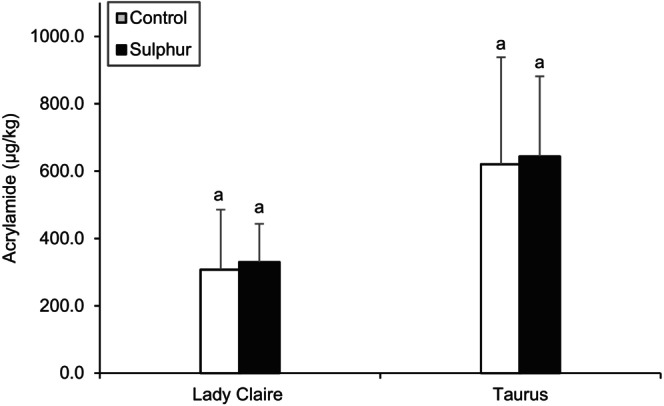
Acrylamide content of potato crisps, effect of sulfur fertilization. Concentrations are expressed in μgkg^−1^. Different letters indicate significant differences (*P* < 0.05) from controls within the same cultivar. Results are expressed as means ± SDs for *n* = 24.

Figure 1 shows acrylamide content of potato crisps from raw potatoes grown with and without sulfur.

No significant difference in acrylamide levels was measured in crisps from both varieties when comparing sulfur grown and controls. Lady Claire crisps had overall low levels of acrylamide, 329.7 ± 113.8 and 307.4 ± 178.3 μg kg^−1^ in sulfur and controls respectively, which fall below the benchmark level of 750 μg kg^−1^ specified by European Commission Regulation (EU) 2017/2158^54^). Higher acrylamide content was measured in potato crisps from the Taurus cultivar in controls (643.7 ± 238.0 μg kg^−1^) and in sulfur samples (620.5 ± 317.7 μg kg^−1^). Higher levels of reducing sugars in raw potatoes, and consequently higher content of acrylamide in the crisps, have often been observed in the Taurus cultivar compared with the Lady Claire cultivar.^17^, ^52^, ^53^, ^55^

Overall, these results are in line with previous studies where sulfur nutrition generally led to lower total amino acids and sugars or glucose levels, but not necessarily lower acrylamide formation; these effects were also found to be variety dependent as in previous studies.^19^, ^20^, ^21^

### Basalt rock aggregate application

In the first trial, which started in August, potato tubers had to be harvested after 12 weeks, 1 month sooner than usual, due to the weather becoming too cold. This resulted in small tubers with an average weight of 39.3 ± 10.2 g in Lady Claire controls, 35.8 ± 7.0 g in Lady Claire with BA, 36.9 ± 8.2 g in Taurus controls, and 40.1 ± 9.2 g in Taurus with BA (Supporting Information, Table S6). The addition of BA did not affect the weight of the tubers as no statistical difference was found in comparison with the controls. The second trial was conducted in season, with tubers planted in April and harvested in July. As in the first trial, the addition of BA during growth did not affect the weight of the harvested tubers. The average weight of Lady Claire was 124.8 ± 42.5 g and 98.6 ± 18.6 g in the BA condition and controls respectively, 116.6 ± 17.1 g and 134.4 ± 25.0 g in Taurus potatoes (Supporting Information, Table S6).

Table 2 shows levels of sugars and amino acids from the first trial and Table 3 shows the levels from the second trial.

**Table 2:** Sugar and amino acidic profile of raw potato tubers grown with and without application of volcanic rock dust, first trial^a^

Variety	Lady Claire		Taurus	
Treatment^b^	Control	Volcanic rock	Control	Volcanic rock
Glucose	12.94 ± 5.90 a	8.87 ± 5.31 b	6.79 ± 3.58 a	11.32 ± 6.28 b
Fructose	1.02 ± 0.29 a	0.95 ± 0.25 a	2.15 ± 0.48 a	2.41 ± 0.92 a
**Total reducing sugars**	**14.20 ± 6.05 a**	**10.15 ± 6.11 b**	**8.92 ± 4.22 a**	**13.86 ± 7.18 b**
Sucrose	12.13 ± 0.96 a	10.80 ± 1.17 b	14.37 ± 2.49 a	13.33 ± 2.31 a
Asparagine	1.30 ± 0.53 a	0.91 ± 0.37 b	1.03 ± 0.43 a	1.11 ± 0.50 a
Alanine	0.74 ± 0.48 a	0.54 ± 0.10 a	0.50 ± 0.17 a	0.46 ± 0.11 a
Valine	2.61 ± 0.94 a	2.41 ± 0.65 a	1.72 ± 0.38 a	2.12 ± 0.52 b
Isoleucine	1.04 ± 0.36 a	1.24 ± 0.39 a	0.92 ± 0.24 a	1.11 ± 0.34 b
Glycine	0.81 ± 0.14 a	0.70 ± 0.05 a	0.70 ± 0.28 a	0.74 ± 0.09 a
Serine	0.50 ± 0.18 a	0.50 ± 0.08 a	0.34 ± 0.06 a	0.43 ± 0.10 b
Threonine	0.84 ± 0.26 a	0.96 ± 0.26 a	0.86 ± 0.24 a	1.11 ± 0.27 b
Glutamine	n.d.	n.d.	0.42 ± 0.03 a	0.32 ± 0.14 a
Lysine	0.95 ± 0.46 a	1.12 ± 0.49 a	0.86 ± 0.32 a	1.06 ± 0.43 a
Tyrosine	0.66 ± 0.24 a	0.91 ± 0.38 b	0.73 ± 0.23 a	0.81 ± 0.30 a
Tryptophan	1.26 ± 0.30 a	1.44 ± 0.47 a	1.41 ± 0.40 a	1.57 ± 0.50 a
**Total amino acids**	**9.76 ± 3.81 a**	**10.35 ± 3.43 a**	**8.07 ± 2.37 a**	**10.51 ± 3.40 a**

^a^ Concentrations are expressed in μgmg^−1^ cycloleucine equivalents. Results are expressed as means ± SDs, *n* = 24. n.d.: not detected.

^b^ Different letters in the same row indicate significant differences (*P* < 0.05) between treated samples and controls within the same cultivar.

**Table 3:** Sugar and amino acidic profile of raw potato tubers grown with and without application of volcanic rock dust, second trial^a^

Variety	Lady Claire		Taurus	
Treatment^b^	Control	Volcanic rock	Control	Volcanic rock
Glucose	2.24 ± 0.37 a	2.19 ± 0.35 a	11.53 ± 4.88 a	11.96 ± 8.50 a
Fructose	1.22 ± 0.40 a	1.01 ± 0.26 a	6.59 ± 2.99 a	5.96 ± 4.40 a
**Total reducing sugars**	**3.46 ± 0.75 a**	**3.36 ± 0.76 a**	**18.12 ± 7.42 a**	**19.32 ± 14.80 a**
Sucrose	16.93 ± 2.43 a	19.28 ± 3.72a	32.25 ± 2.69 a	33.58 ± 6.50 a
Asparagine	3.82 ± 0.87 a	5.17 ± 1.95 b	2.86 ± 0.99 a	1.98 ± 1.67 a
Alanine	0.80 ± 0.15 a	1.33 ± 0.57 b	1.25 ± 0.56 a	1.66 ± 0.47 a
Valine	1.93 ± 0.34 a	2.33 ± 0.50 b	3.36 ± 0.61 a	4.15 ± 0.94 b
Isoleucine	0.92 ± 0.27 a	1.16 ± 0.41 a	2.14 ± 0.52 a	2.43 ± 0.59 a
Proline	0.24 ± 0.09 a	0.78 ± 0.69 a	0.55 ± 0.18 a	0.73 ± 0.36 a
Glycine	0.63 ± 0.10 a	0.76 ± 0.21 a	1.28 ± 0.30 a	1.71 ± 0.35 b
Serine	0.62 ± 0.15 a	1.04 ± 0.29 b	1.03 ± 0.24 a	1.12 ± 0.29 a
Threonine	0.99 ± 0.14 a	1.25 ± 0.26 b	2.45 ± 0.51 a	2.71 ± 0.62 a
Aspartic acid	1.21 ± 0.12 a	3.24 ± 1.90 a	4.09 ± 0.67 a	5.33 ± 1.06 b
Glutamic acid	2.68 ± 0.39 a	2.93 ± 0.50 a	3.85 ± 0.54 a	5.50 ± 0.43 b
Lysine	0.54 ± 0.14 a	0.64 ± 0.24 a	0.79 ± 0.25 a	0.75 ± 0.34 a
Tyrosine	0.73 ± 0.30 a	0.82 ± 0.36 a	1.65 ± 0.41 a	1.49 ± 0.73 a
Tryptophan	1.31 ± 0.35 a	1.21 ± 0.31 a	3.13 ± 1.19 a	2.76 ± 1.57 a
**Total amino acids**	**14.71 ± 1.27 a**	**19.53 ± 5.45 b**	**27.93 ± 7.60 a**	**26.14 ± 11.08 a**

^a^ Concentrations are expressed in μgmg^−1^ cycloleucine equivalent. Results are expressed as means ± SDs, *n* = 12.

^b^ Different letters in the same row indicate significant differences (*P* < 0.05) between treated samples and controls within the same cultivar.

In the first trial potatoes from cultivar Lady Claire, grown with BA added, exhibited significantly lower levels of glucose (8.87 ± 5.31 μg mg^−1^) in comparison with controls (12.94 ± 5.90 μg mg^−1^); they also had lower levels of sucrose (10.80 ± 1.17 μg mg^−1^ in BA; 12.13 ± 0.96 μg mg^−1^ in controls), and a lower overall total reducing sugar content (10.15 ± 6.11 μg mg^−1^ in BA; 14.20 ± 6.05 μg mg^−1^ in controls). The amino acidic profile was not significantly different from controls, although there was lower asparagine and higher tyrosine content in tubers grown with BA. Potatoes from the Taurus cultivar in the first trial, grown with BA, had significantly higher glucose levels (11.32 ± 6.28 μg mg^−1^) than controls (6.79 ± 3.58 μg mg^−1^) and higher total reducing sugar content (13.86 ± 7.18 μg mg^−1^ in BA; 8.92 ± 4.22 μg mg^−1^ in controls). These tubers also showed higher values of valine, isoleucine, serine, and threonine. No variations in the sugar content were observed in potatoes from both cultivars in the second trial when grown with BA (Table 3).

Considering both growing conditions, with and without BA, Lady Claire tubers exhibited significantly lower levels of glucose (2.19 ± 0.35 μg mg^−1^ and 2.24 ± 0.37 μg mg^−1^ respectively), fructose (1.01 ± 0.26 μg mg^−1^; 1.22 ± 0.40 μg mg^−1^) and sucrose (19.28 ± 3.72 μg mg^−1^; 16.93 ± 2.43 μg mg^−1^) than Taurus (glucose: 11.96 ± 8.50 μg mg^−1^, 11.53 ± 4.88 μg mg^−1^; fructose: 5.96 ± 4.40 μg mg^−1^, 6.59 ± 2.99 μg mg^−1^; sucrose: 33.58 ± 6.50 μg mg^−1^, 32.25 ± 2.69 μg mg^−1^) and also compared to Lady Claire from the first trial (Table 2). Tubers from the Taurus cultivar often contain higher levels of reducing sugars and had greater variability than Lady Claire.^17^, ^52^, ^53^ The higher sugar content in Lady Claire potatoes from the first trial in comparison with the second can be explained by the fact that tubers were harvested before they reached full maturity.^56^ Looking at the amino acid profile of Lady Claire from the second trial, potatoes grown with BA show a higher total amino acid content (19.53 ± 5.45 μg mg^−1^) than controls (14.71 ± 1.27 μg mg^−1^), with a higher content of asparagine, alanine, valine, serine, and threonine. Taurus tubers grown with BA show higher levels of valine, glycine, aspartic acid, and glutamic acid.

**Figure 2:**
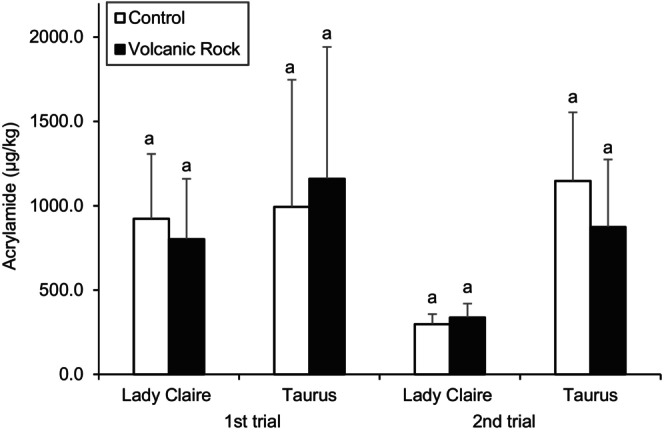
Acrylamide content of potato crisps, effect of basalt rock aggregate application. Concentrations are expressed in μgkg^−1^. Different letters indicate significant differences (*P* < 0.05) from controls within the same cultivar. Results are expressed as means ± SDs; *n* = 24 for the first trial, and *n* = 12 for the second trial.

Figure 2 shows the acrylamide content of potato crisps from the first and second trials. No significant difference in acrylamide levels were observed, in both trials and cultivars, as a consequence of growing tubers with addition of BA when compared with controls. Lady Claire crisps from the second trial contained significantly lower acrylamide (337.0 ± 83.0 μg kg^−1^ with BA; 298.0 ± 59.5 μg kg^−1^ controls) than crisps of the same cultivar from the first trial (801.8 ± 357.8 μg kg^−1^ with BA; 922.6 ± 384.5 μg kg^−1^ controls), and also in comparison with crisps from the Taurus cultivar from both trials (first trial: 1160.1 ± 781.7 μg kg^−1^ with BA, 993.4 ± 754.0 μg kg^−1^ controls; second trial: 873.8 ± 400.8 μg kg^−1^ with BA, 1147.4 ± 406.9 μg kg^−1^ controls), which is in accordance with reducing sugar levels in the raw material (Table 2). It can be observed that, in the first trial the variability of both reducing sugar content (Table 2) and acrylamide levels (Fig. 2) is very high, which is reflected in the high standard deviation of the results. This can be due to the seasonality effect, as mentioned above, and can explain why the higher reducing sugar content in the Lady Claire controls and the Taurus BA group, did not result in significantly higher acrylamide in the crisps.

Overall, the two trials showed no changes in plant growth, crop yield (data not shown), and tuber weight when potatoes were grown in pots with addition of BA, and no negative effects were observed. When a difference was found in the nutritional profile of the tubers, including reducing sugars content, this did not affect the acrylamide formation in potato crisps and consequently the suitability of the tubers for the processing market. No consistent trend was found in variations related to the sugar or amino acidic profile of the potatoes grown with BA in comparison with their controls. This suggests that any difference observed is most likely attributable to variability among tubers, cultivars, and growing seasons rather than to the addition of BA to the soil.

### Speed breeding under LED lights

Table 4 summarizes key indicators of plant growth and lifecycle, measured during the trial conducted to compare speed breeding under LED lights and glasshouse growing of potato plants from three different cultivars.

**Table 4:** Indicators of plant growth and lifecycles for plants grown under LED lights and glasshouse controls

Variety	Treatment	First shots (week)	Buds appearing (week)	Flowers opening (week)	Harvest time (week)	Final height (cm)	Tuber average weight (g)^a^
Lady Claire	Control	5	9	10	20	80–90	85.5 ± 15.1 a
	LED lights	4	7	8	14	50–60	79.1 ± 14.5 a
Taurus	Control	6	10	12	20	80–90	80.3 ± 16.3 a
	LED lights	6	9	10	16	50–60	89.3 ± 15.0 a
Desiree	Control	4	8	10	20	80–90	119.5 ± 37.4 a
	LED lights	3	7	8	14	50–60	118.6 ± 27.0 a

^a^ Results are expressed as means ± SDs, *n* = 12. Different letters indicate a significant difference (*P* < 0.05) between different growing conditions within the same cultivar.

As expected, speed breeding resulted in earlier first signs of shoots for Desiree and Lady Claire potato plants, and earlier appearing of flower buds and earlier flower opening for plants of all three varieties, in comparison with controls grown in the glasshouse, as well as early harvest. Unexpectedly, the final average height of plants was lower (50–60 cm) in all three varieties subjected to speed breeding in comparison with glasshouse‐grown plants (80–90 cm). When looking at average weights of potato tubers, no significant difference was observed between plants grown under LED lights and controls, in all three cultivars.

Table 5 shows the sugar profile, and Table 6 shows the amino acid profile, of raw tubers from the Lady Claire, Taurus, and Desiree cultivars, when grown under LED lights in comparison with their glasshouse controls.

**Table 5:** Sugar profile of raw potato tubers grown under LED lights and glasshouse controls^a^

Variety	Lady Claire		Taurus		Desiree	
Treatment^b^	Control	LED lights	Control	LED lights	Control	LED lights
Glucose	3.77 ± 0.57a	6.41 ± 1.43 b	4.30 ± 1.27 a	16.18 ± 2.78 b	15.11 ± 5.92 a	28.52 ± 12.85 b
Fructose	1.28 ± 0.47a	3.32 ± 2.00 b	1.91 ± 0.80 a	13.79 ± 3.82 b	12.93 ± 6.85 a	23.00 ± 10.03 b
**Total reducing sugars**	**4.83 ± 0.48 a**	**9.34 ± 2.71 b**	**6.21 ± 1.93 a**	**30.76 ± 2.84 b**	**28.04 ± 12.37 a**	**51.52 ± 22.82 b**
Sucrose	14.08 ± 1.60 a	17.44 ± 2.04 b	21.34 ± 3.40 a	19.25 ± 2.60 a	28.54 ± 7.39 a	28.30 ± 5.32 a

^a^ Concentrations are expressed in μgmg^−1^ cycloleucine equivalent. Results are expressed as means ± SDs, *n* = 12.

^b^ Different letters in the same row indicate significant differences (*P* < 0.05) between different growing conditions within the same cultivar.

**Table 6:** Amino acidic profile of raw potato tubers grown under LED lights and glasshouse controls^a^

Variety	Lady Claire		Taurus		Desiree	
Treatment^b^	Control	LED lights	Control	LED lights	Control	LED lights
Asparagine	1.41 ± 0.87 a	1.74 ± 0.90 a	1.31 ± 0.60 a	1.34 ± 0.81 a	1.32 ± 0.58 a	2.23 ± 0.95 b
Alanine	0.98 ± 0.17 a	0.76 ± 0.14 b	1.26 ± 0.20 a	0.99 ± 0.29 a	1.28 ± 0.31 a	1.02 ± 0.34 a
Valine	1.82 ± 0.44 a	1.98 ± 0.75 a	3.03 ± 0.50 a	4.09 ± 0.81 b	4.46 ± 0.75 a	5.25 ± 1.26 a
Isoleucine	0.75 ± 0.31 a	1.04 ± 0.48 a	1.81 ± 0.31 a	2.67 ± 0.66 b	2.99 ± 0.62 a	3.59 ± 0.95 a
Proline	n.d.	n.d.	0.23 ± 0.05	n.d.	n.d.	n.d.
Glycine	0.60 ± 0.10 a	0.68 ± 0.04 a	0.95 ± 0.39 a	1.12 ± 0.29 a	1.16 ± 0.33 a	1.19 ± 0.32 a
Serine	0.57 ± 0.13 a	0.51 ± 0.16 a	0.81 ± 0.21 a	1.15 ± 0.32 b	0.99 ± 0.18 a	1.00 ± 0.23 a
Threonine	1.18 ± 0.19 a	1.02 ± 0.29 a	1.88 ± 0.45 a	2.12 ± 0.35 a	2.52 ± 0.44 a	2.85 ± 0.77 a
Aspartic acid	1.62 ± 0.15 a	1.81 ± 0.33 a	3.06 ± 1.45 a	1.56 ± 0.57 a	1.94 ± 0.98 a	3.14 ± 0.24 a
Glutamic acid	2.39 ± 0.51 a	2.75 ± 0.47 a	3.04 ± 0.64 a	3.24 ± 0.52 a	2.98 ± 0.89 a	3.75 ± 1.04 a
Phenylalanine	n.d.	n.d.	n.d.	n.d.	2.08 ± 0.26 a	1.81 ± 0.49 a
Lysine	n.d.	n.d.	0.34 ± 0.08	n.d.	0.77 ± 0.27 a	1.39 ± 0.35 b
Tyrosine	n.d.	n.d.	1.12 ± 0.30 a	0.64 ± 0.15 b	2.75 ± 0.61 a	4.77 ± 1.43 b
Tryptophan	1.34 ± 0.35 a	1.79 ± 0.37 b	2.70 ± 0.52 a	2.35 ± 0.34 a	2.82 ± 0.70 a	4.29 ± 1.22 b
**Total amino acids**	**11.53 ± 2.51 a**	**12.36 ± 1.71 a**	**16.75 ± 2.77 a**	**19.64 ± 3.49 b**	**25.22 ± 3.44 a**	**31.14 ± 4.57 b**

^a^ Concentrations are expressed in μgmg^−1^ cycloleucine equivalents. Results are expressed as means ± SDs, *n* = 12. n.d.: not detected.

^b^ Different letters in the same row indicate significant differences (*P* < 0.05) between different growing conditions within the same cultivar.

For all three varieties of potatoes, speed breeding under the selected conditions led to a significantly higher content in both reducing sugars. For Lady Claire, the glucose content in potatoes grown with LED lights was 6.41 ± 1.43 μg mg^−1^ in comparison with 3.77 ± 0.57 μg mg^−1^ in controls with a 70.02% increase; fructose levels were 3.32 ± 2.00 μg mg^−1^ in LED lights, 159.37% higher than controls with 1.28 ± 0.47 μg mg^−1^. Lady Claire LED lights also showed significantly higher sucrose content than controls. In Taurus tubers the increase in glucose and fructose was found to be 276.28% and 620.42% respectively, with 16.18 ± 2.78 μg mg^−1^ glucose and 13.79 ± 3.82 μg mg^−1^ fructose in potatoes grown under LED lights, compared with controls with 4.30 ± 1.27 μg mg^−1^ glucose and 1.91 ± 0.80 μg mg^−1^ fructose. Tubers from the cultivar Desiree showed the highest sugars content overall, again with a significant increase when grown under LED lights (88.75% more glucose and 77.88% more fructose than glasshouse controls). Glucose and fructose content were 28.52 ± 12.85 μg mg^−1^ and 23.00 ± 10.03 μg mg^−1^ respectively in LED lights, 15.11 ± 5.92 μg mg^−1^ and 12.93 ± 6.85 μg mg^−1^ in controls. No consistent trend was observed in variations in the amino acidic profile, with some changes found across the different cultivars. Lady Claire showed lower alanine and higher tryptophan when grown with LED lights compared with the glasshouse plants. Taurus grown under LED lights had higher valine, isoleucine, serine, and lower tyrosine than controls. Proline and lysine were only detected in control potatoes. The total amount of amino acids was found to be higher in Taurus grown under LED lights than in the glasshouse (19.64 ± 3.49 μg mg^−1^ and 16.75 ± 2.77 μg mg^−1^ respectively). Potatoes from the Desiree cultivar grown under LED lights showed higher asparagine, lysine, tyrosine, and tryptophan content than controls, as well as higher total amino acids (31.14 ± 4.57 μg mg^−1^ and 25.22 ± 3.44 μg mg^−1^ respectively).

**Figure 3:**
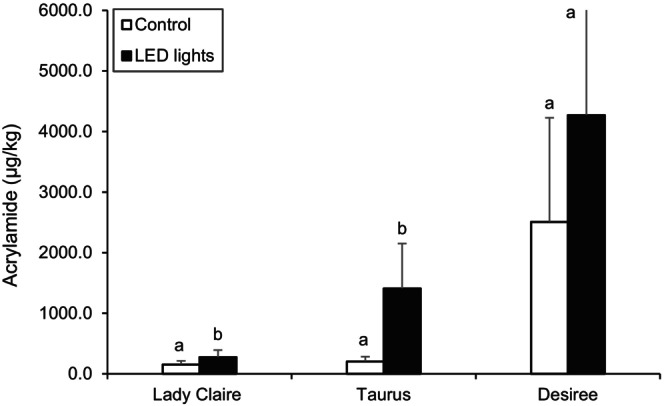
Acrylamide content of potato crisps, effect of speed breeding under LED lights. Concentrations are expressed in μgkg^−1^. Different letters indicate significant differences (*P* < 0.05) from controls within the same cultivar. Results are expressed as means ± SDs for *n* = 12.

Figure 3 shows the acrylamide content in potato crisps generated from tubers of the three cultivars grown with speed breeding and glasshouse controls.

As expected from the levels of reducing sugars measured in the raw potatoes, it was found that growth under LED light, in the tested conditions, led to crisps with higher levels of acrylamide in all three tested varieties. Crisps from Lady Claire potatoes showed low acrylamide levels overall, below the benchmark level of 750 μg kg^−1^, with an average of 153.19 ± 60.14 μg kg^−1^ of acrylamide in controls, and significantly higher levels in the LED lights samples, 274.06 ± 117.87 μg kg^−1^, which corresponds to a 78.90% increase. The measured acrylamide content of crisps from the Taurus cultivar was also very low in glasshouse controls, 203.43 ± 80.32 μg kg^−1^, and significantly higher in tubers from plants grown under LED lights, 1408.90 ± 741.52 μg kg^−1^ corresponding to 592.58% increase. The Desiree cultivar produced crisps with the overall highest levels of acrylamide, 2508.13 ± 1718.70 μg kg^−1^ in controls, and 4269.97 ± 2714.40 μg kg^−1^ under LED lights (70.25% increase), which reflected the sugar content of the raw potatoes.

As far as the authors of the current study are aware, this is the first study that has considered directly the impact of growing potato plants under LED lights irradiation on acrylamide formation in potato crisps. These results suggest that speed breeding in the tested conditions of LED lighting system is not suitable as an alternative to traditional glasshouse growing under natural lighting, especially for potatoes destined for the processing market, where low reducing sugars levels are an essential requirement.

The observed increase in reducing sugars, and consequently acrylamide formation, could be linked to the ratio between red and blue lights used in the LED lighting system. The photosynthetic pigments in plants absorb light more strongly within the blue (400–500 nm) and red (600–700 nm) spectra. These wavelengths are the most effectively used during photosynthesis and are often chosen for plant growth in systems such as those with LED lights.^57^, ^58^ Several studies have investigated the role of both red and blue light in photosynthesis and their respective influence on different aspects of plant growth, plant health and crop yield. Interestingly, Pundir *et al*. found that a monochromatic red LED spectrum and a 70:30 red:blue combination of LED light induced accumulation of carbohydrates in leaves and tubers of potato plants, whereas a reduction in carbohydrates was found with the opposite LED light conditions of monochromatic blue or a 30:70 red:blue ratio.^48^ These findings are in line with the results obtained in the current study applying similar LED lighting ratios of red and blue light (Supporting Information, Figs S2–S4).

## CONCLUSIONS

This study highlighted the importance of examining the effects of agronomic practices and growing conditions on crop quality and safety, as these are the primary factors influencing nutritional composition. This approach also offers a cost‐effective means of addressing changes in crop nutritional profiles.

The relationship between sulfur supply and amino acid biosynthesis in potatoes, which varies by cultivar, is a key consideration for the production of tubers produced with the aim of processing, potentially helping to reduce acrylamide precursors.

The effects of BA application to soil during crop growth and the use of speed breeding under LED lights were investigated for the first time, focusing on their impact on nutritional profiles, acrylamide formation, and reducing sugar content, specifically in relation to processing suitability.

The addition of BA had no significant effect on the nutritional values of potatoes, which is a positive outcome, demonstrating an additional environmental benefit. Rock dust contributes to soil inorganic carbon accumulation, promoting carbon sequestration without adversely impacting crop growth, quality, or safety.

These preliminary studies confirm the importance of conducting future research on BA application, including field trials, other crops, different soil types. Further studies should be conducted to clarify the role of different LED lighting conditions not only on plant growth and crop yield but also on crops nutritional profile. This could potentially reveal beneficial settings of LED lights, which might decrease sugar accumulation.

## Supporting information


**Figure S1.** Sulphur field trial set‐up.

**Figure S2.** LED lights spectrum readings, Lady Claire.

**Figure S3.** LED lights spectrum readings, Taurus.

**Figure S4.** LED lights spectrum readings, Desiree.

**Table S5.** Average weights of potatoes from sulphur trials.

**Table S6.** Average weights of potatoes from volcanic rock dust trials.

## Data Availability

The data that support the findings of this study are available from the corresponding author upon reasonable request.
