# Supplementary figures and images for: Effects of agronomical practices on potato growth, nutritional profile, and suitability for frying
Source: J Sci Food Agric. 2025 Jan 30;105(7):3983–92. doi: 10.1002/jsfa.14147 (PMC11990047; doi:10.1002/jsfa.14147)

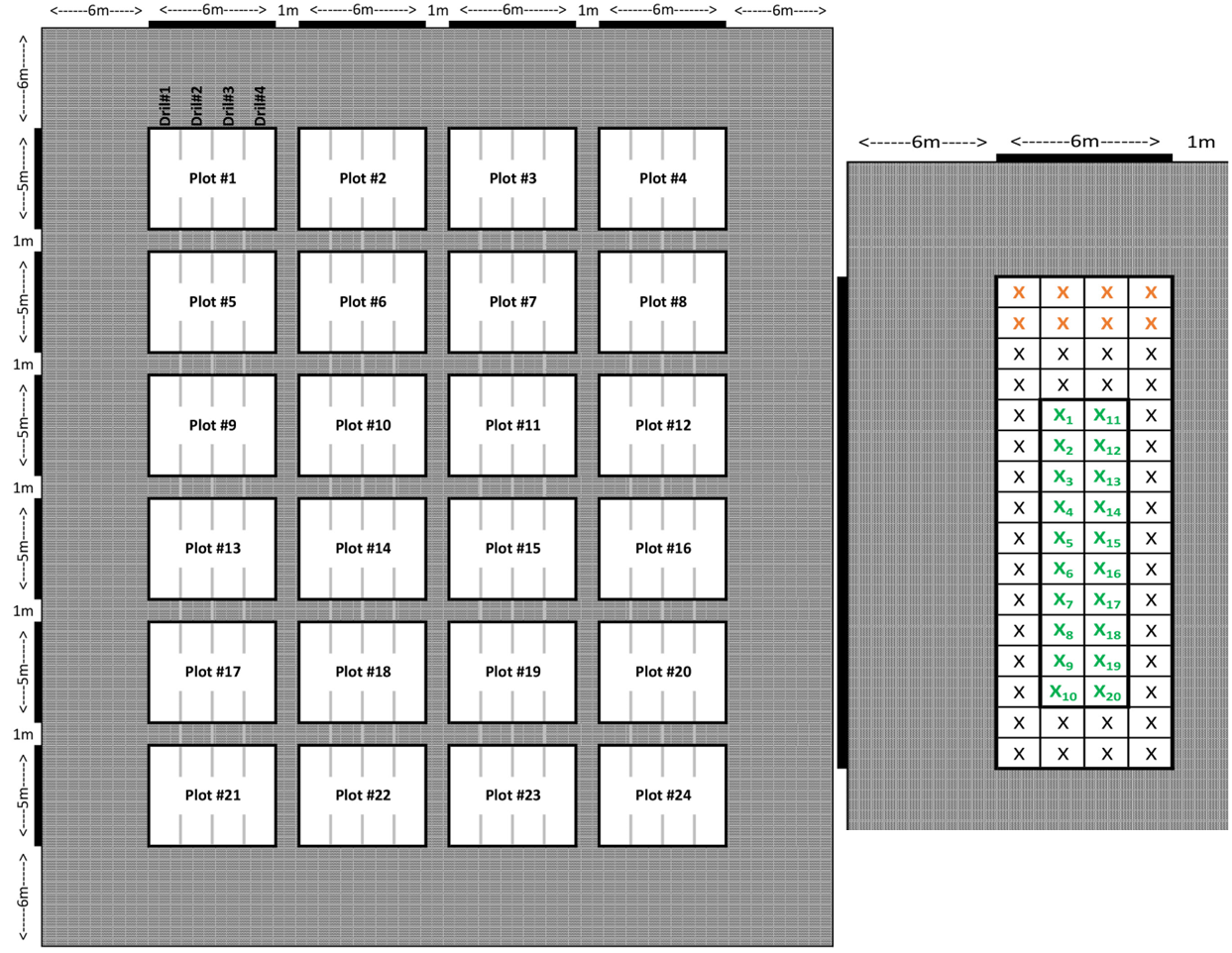

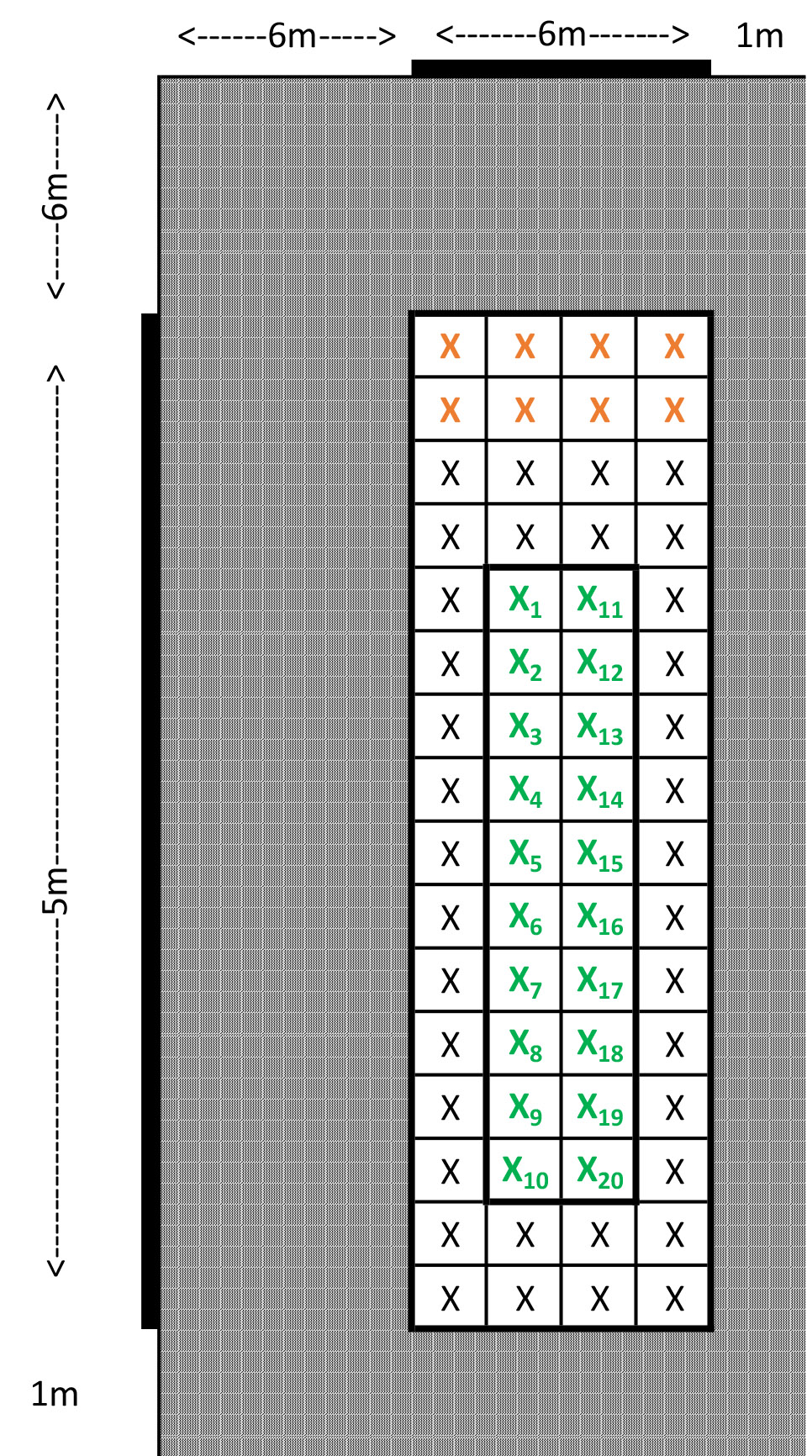


Figure S1 - Sulphur field trial set-up

Supplement: Supplementary file 1 — Figure S1. Sulphur field trial set‐up. [file JSFA-105-3983-s006.docx]

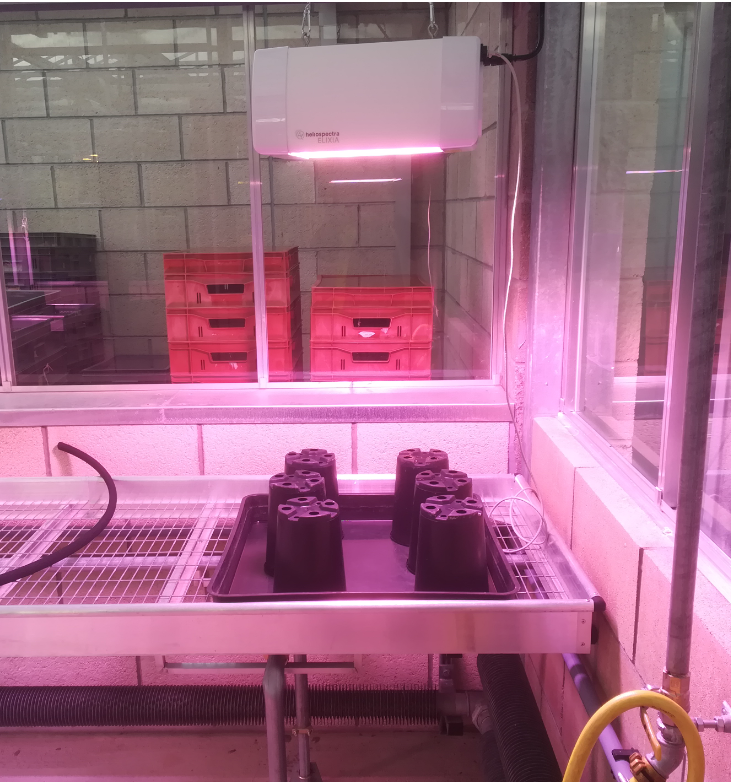

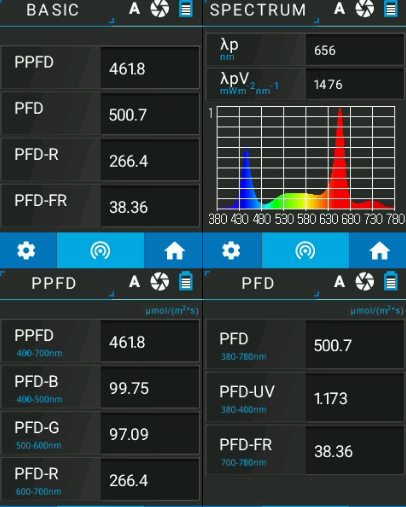


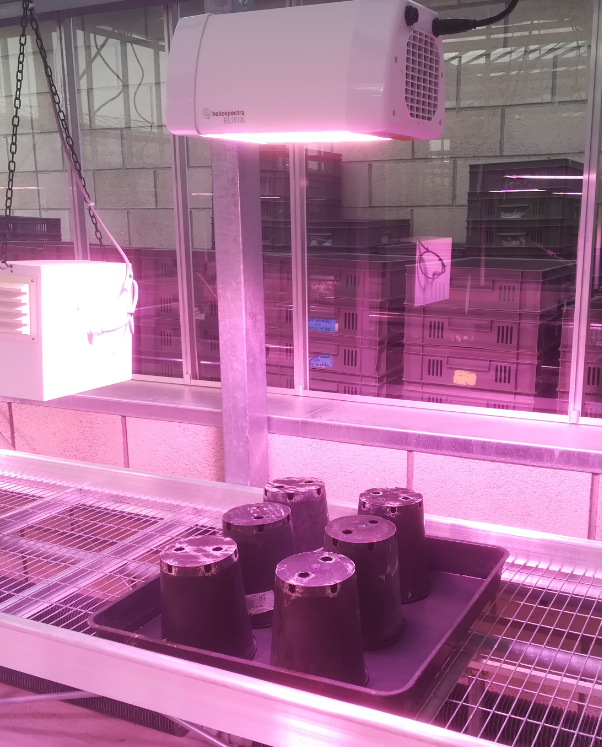

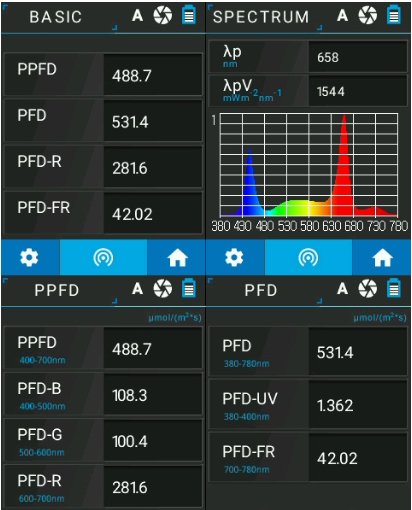


Figure S2 – LED lights spectrum readings, Lady Claire.

Reading 5

Reading 5

Supplement: Supplementary file 2 — Figure S2. LED lights spectrum readings, Lady Claire. [file JSFA-105-3983-s002.docx]

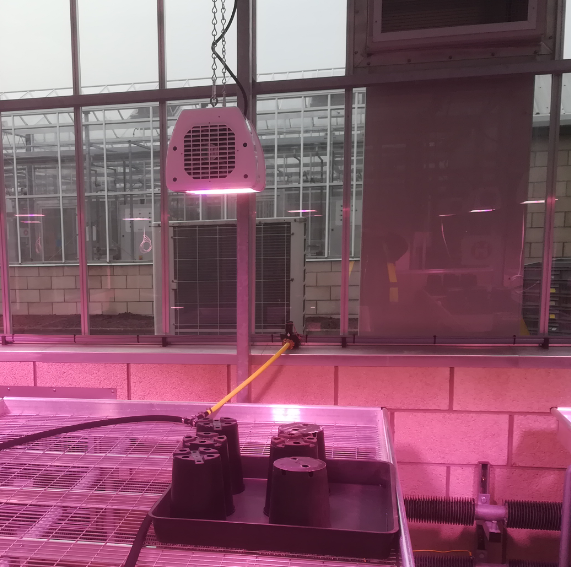

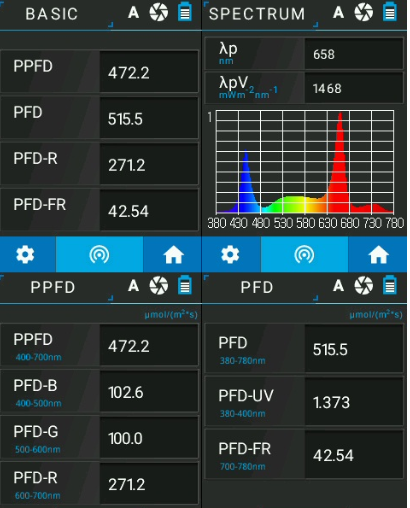

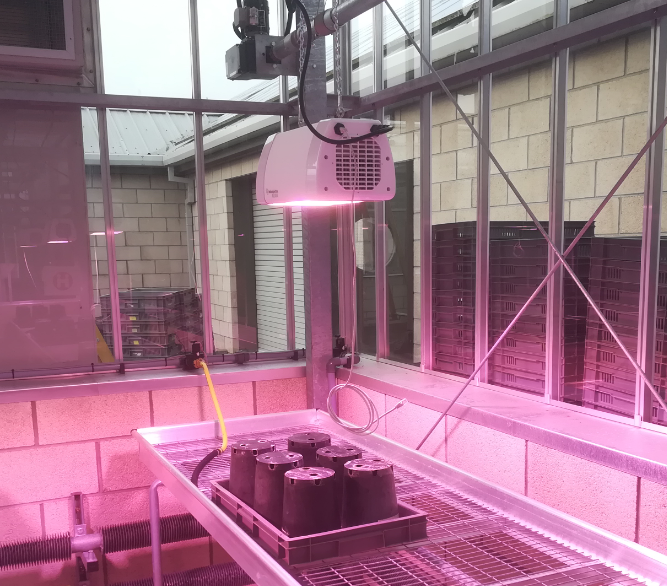

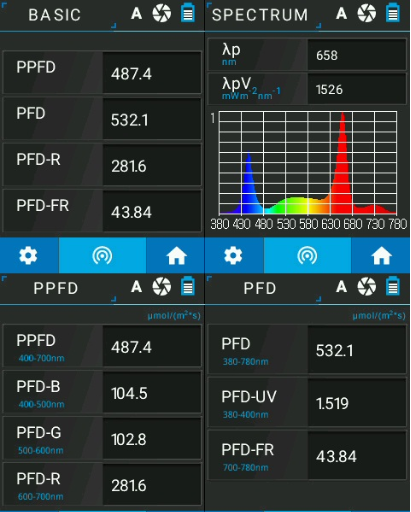


Figure S3 - LED lights spectrum readings, Taurus

Supplement: Supplementary file 3 — Figure S3. LED lights spectrum readings, Taurus. [file JSFA-105-3983-s001.docx]

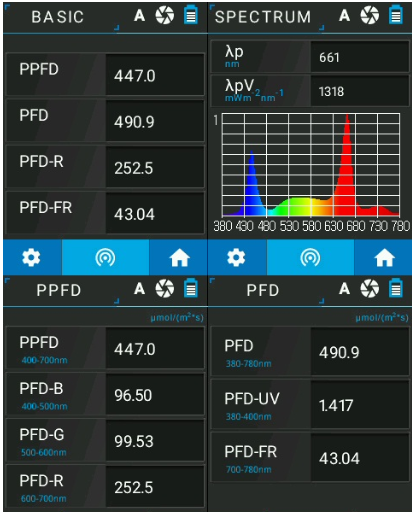

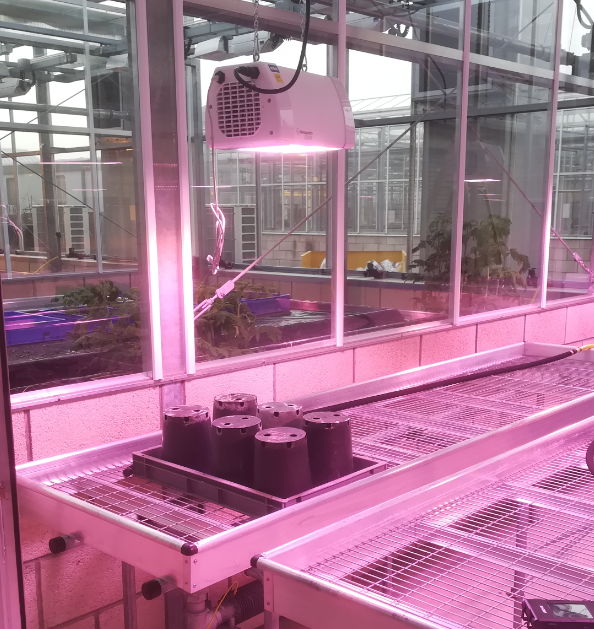

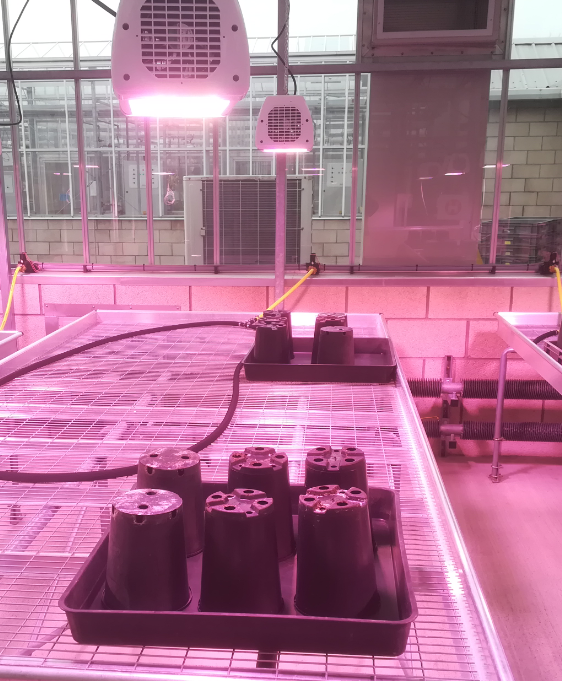

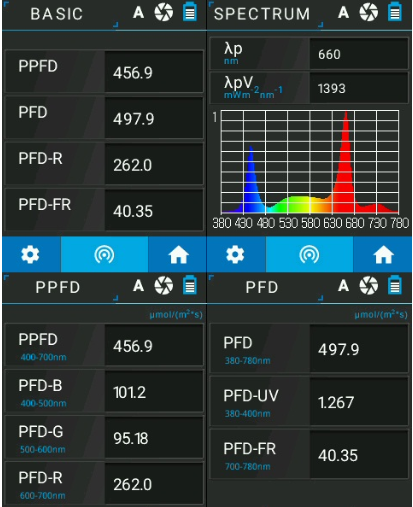


Figure S4 – LED lights spectrum readings, Desiree

Supplement: Supplementary file 4 — Figure S4. LED lights spectrum readings, Desiree. [file JSFA-105-3983-s005.docx]
