# Supplementary material for: Effects of agronomical practices on potato growth, nutritional profile, and suitability for frying
Source: J Sci Food Agric. 2025 Jan 30;105(7):3983–92. doi: 10.1002/jsfa.14147 (PMC11990047; doi:10.1002/jsfa.14147)
Supplement: Supplementary file 5 — Table S5. Average weights of potatoes from sulphur trials. [file JSFA-105-3983-s004.docx]

Table S5 – Average weights of potatoes from sulphur trials.

| **Variety** | **Treatment** | **Weights (g)^[[1]](#footnote-1)^** |
| --- | --- | --- |
| Lady Claire | Control | 201.5 ± 30.3 a |
|  | Sulphur | 179.6 ± 29.3 b |
| Taurus | Control | 171.6 ± 18.9 a |
|  | Sulphur | 183.4 ± 18.4 b |

1. Different letters indicate a significant difference (p < 0.05) between different growing conditions within the same cultivar. Results are expressed as mean ± SD, n = 24 [↑](#footnote-ref-1)
