# Supplementary material for: Effects of agronomical practices on potato growth, nutritional profile, and suitability for frying
Source: J Sci Food Agric. 2025 Jan 30;105(7):3983–92. doi: 10.1002/jsfa.14147 (PMC11990047; doi:10.1002/jsfa.14147)
Supplement: Supplementary file 6 — Table S6. Average weights of potatoes from volcanic rock dust trials. [file JSFA-105-3983-s003.docx]

Table S6 – Average weights of potatoes from volcanic rock dust trials^[[1]](#footnote-1)^.

| **Variety** | **Treatment** | **1^st^ trial** | **2^nd^ trial** |
| --- | --- | --- | --- |
| Lady Claire | Control | 39.3 ± 10.2 a | 98.6 ± 18.6 a |
|  | Volcanic Rock | 35.8 ± 7.0 a | 124.8 ± 42.5 a |
| Taurus | Control | 36.9 ± 8.2 a | 134.4 ± 25.0 a |
|  | Volcanic Rock | 40.1 ± 9.2 a | 116.6 ± 17.1 a |

1. Different letters indicate a significant difference (p < 0.05) between different growing conditions within the same cultivar and trial. Results are expressed as mean ± SD, n = 24 for the 1^st^ trial, n = 12 for the 2^nd^ trial. [↑](#footnote-ref-1)
